# Chemical crystallography and crystal engineering

**DOI:** 10.1107/S2052252514021976

**Published:** 2014-10-10

**Authors:** Gautam R. Desiraju

**Affiliations:** a Solid State and Structural Chemistry Unit, Indian Institute of Science, Bangalore 560 012, India

**Keywords:** Editorial, chemical crystallography, crystal engineering

## Abstract

Today, there is very little doubt that chemistry owes as much to crystallography as crystallography does to chemistry. This mutual synergy defines modern chemical crystallography.

Ever since it was shown that the crystal structure of NaCl could be determined from the diffraction pattern of the substance, the subject of chemistry has been inextricably linked with crystallography. While the accuracy of the molecular parameters obtained from a crystallographic study are generally comparable with those obtained from spectroscopy or computation, the generality and ease of application of crystallographic methods ensured their indispensability in structural chemistry. All that is required today is a single-crystal of the compound under consideration, a standard diffractometer and a computer. Various types of bonding – ionic, covalent, electron deficient, quadruple, organometallic and the so-called non-covalent bonds, like the hydrogen bond, the metallic bond and more lately, the halogen bond – could only have been understood if a method existed that allowed for the very accurate measurement of molecular and intermolecular geometrical parameters in a variety of compounds. Any development in solid-state chemistry obviously predicates a thorough knowledge of crystal structure and the momentous progress in this field owes much to the determination of structures that span the range from silicates and minerals, to complex oxides, perovskite superconductors, zeolites and catalysts right through to our present day appreciation of aperiodic crystals.

But going beyond structure to dynamics and synthesis, the biggest stumbling block to a more general acceptance of crystallography in mainstream chemistry was that chemists, until say the 1980s, were skeptical about the relevance of a molecular structure obtained from the solid state in any consideration of reactions and dynamic processes that are usually studied in solution. ‘What is the relevance of a solid-state structure to solution processes?’ became a rhetorical question that bedeviled the chemical crystallographer. Stubbornly, chemists persisted with solution NMR as *the* method of choice for structure assignment despite the big advances in crystallography itself that allowed say the determination of the absolute configuration of a chiral molecule, the easy and eventually routine determination of the crystal structure of a molecule that did not contain a heavy atom, the very accurate determination of hydrogen atom positions with neutron diffraction and the observation of bonding electrons in a molecule or between molecules with charge density methodology. Two developments broke this deadlock: the synthesis of organometallic cluster compounds in the 1980s saw the appearance of a large number of substances whose molecular structures *could not* be determined with solution NMR. Crystallography was identified as the *only* method that could be used to establish the complex topology of metal–metal bonding in these substances. The second milestone, time-resolved crystallography, was facilitated by the availability of synchrotrons and meant that crystals could be studied for short exposure times – shorter than the timescales of some chemical processes. This meant that crystallography had entered the domain of chemical dynamics. Today, there is very little doubt that chemistry owes as much to crystallography as crystallography does to chemistry. This mutual synergy defines modern chemical crystallography.

But what of synthesis? Even as the very first crystal structures of molecular solids were being determined, crystallographers began to ask questions about why certain structures were adopted and others not. Correlations between molecular structure and crystal structure were slow in coming but there was little doubt that this was a problem of fundamental scientific importance: ‘Given the molecular structure of an organic compound, what is its crystal structure?’ Attempts to answer this question computationally using transferable isotropic potentials typically ended in ambiguity and eventually failure because the electrostatic potentials around atoms in crystals are sufficiently variable and anisotropic so that geometrical close packing does not alone determine crystal structure, although it does play a big role. This anisotropy, in fact, leads to the manifestation of characteristic intermolecular interactions which, in turn, lead to directional preferences in the assembly of molecules into crystals. A greater appreciation of these interactions among crystallographers and chemists undoubtedly arose with the growing popularity of supramolecular chemistry in the late 1980s and early 1990s. After this time, any story in chemical crystallography started rather than ended with the determination of the positional parameters *x*, *y* and *z* of all the atoms in the crystal structure. What a structure meant was often more interesting than what it was.

The subject of crystal engineering, or solid-state supramolecular synthesis, deals with the understanding of intermolecular interactions in the context of crystal packing and in applying this understanding to the design of pre-desired crystal structures with specific physical or chemical properties. Analogies between classical organic synthesis and crystal engineering appeared with retrosynthetic analysis being at the crux of the matter. Whether it was the *supramolecular synthon* for molecular solids or the *secondary building unit*, for metal organic framework solids (MOFs), the principle is the same. One can work backwards from the topology of a target crystal structure using directional properties of selected intermolecular interactions to arrive at precursor molecule(s) that yield the desired three-dimensional structure upon crystallization. With the ease of crystal structure determination, crystal engineering quickly went on to become an important subject for study among crystallographers, with database analysis of already published crystal structures becoming a useful auxiliary technique. The study of MOFs became immensely popular because of the wide applicability of these robust compounds in gas storage, catalysis, sensor, photochemical and separation technologies.

Modern crystal engineering includes the study of polymorphism, the phenomenon in which one compound has more than one crystal structure. Polymorphism is of immense importance in the pharmaceutical industry, where different crystalline modifications of a drug can have very different properties and indeed different legal status because they may be eligible for separate patent protection. Co-crystals, or multicomponent molecular crystals, are also being studied actively in the context of the pharmaceutical implications because cocrystallization is an effective technique to change the solubility and dissolution profile of a drug, and hence its bioavailability. Also important today is crystallography in non-ambient conditions, mostly low temperature and high pressure, because there are high chances of obtaining polymorphs under such conditions. Crystal structure prediction is related to polymorphism and has now become a sophisticated computational exercise. We are hence moving towards the definition of a *crystal structure landscape* which is defined by experimental and computationally derived crystal structures of various solid forms of a substance. These forms could include polymorphs, pseudopolymorphs (solvates) and even chemically related congeners. The landscape provides the chemist with a structural profile of the crystallization event, at least in its latter stages, and allows one to address one of the ultimate questions of crystal engineering, namely an elucidation of the mechanism of the supramolecular reaction called crystallization.

Papers in chemical crystallography and crystal engineering have always formed a mainstay in journals of the IUCr from its earliest days. While the basic concepts and principles of crystallography have remained the same, the rapid advances in instrumentation and computing ability have meant that a wider range of problems that span the intersections of wide swathes of crystallography and chemistry are now within the reach and capability of the structural chemist. There is now an urgency to obtain structures of poorly crystalline materials and a concomitant need to be able to examine ever smaller samples. The relevance of electron diffraction, powder methods and the use of synchrotrons and electron lasers will undoubtedly increase in all aspects of chemical crystallography. A subject like crystal engineering has its own specialty journals including those from the Union. The subject is also featured regularly in prominent chemistry journals. The presence of a section on *Chemistry and Crystal Engineering* in **IUCrJ** will only add to all of this. The great volume today of papers of the highest standard in these rapidly growing areas as well as the importance of the open-access concept gives us the confidence to begin this section in the newest journal of the Union.

